# Bedaquiline Resistance in Drug-Resistant Tuberculosis in South Africa: A Systematic Review and Meta-Analysis of Emerging Trends

**DOI:** 10.3390/antibiotics15040385

**Published:** 2026-04-10

**Authors:** Kabelo Gabriel Kaapu, Vukosi Treasure Makondo, Emilyn Costa Conceição, Ivy Rukasha

**Affiliations:** 1 Department of Pathology, School of Medicine, University of Limpopo, Sovenga 0727, Limpopo, South Africa; makondotreasure@gmail.com (V.T.M.); ivy.rukasha@ul.ac.za (I.R.); 2 Division of Molecular Biology and Human Genetics, South African Medical Research Council Centre for Tuberculosis Research, Faculty of Medicine and Health Sciences, Stellenbosch University, Cape Town 7505, Western Cape, South Africa; emilyncosta@sun.ac.za; 3 Department of Microbiology, Polokwane Laboratory, National Health Laboratory Service, Polokwane 0699, Limpopo, South Africa

**Keywords:** Bedaquiline, drug-resistant tuberculosis, *Mycobacterium tuberculosis*, bedaquiline resistance, South Africa, resistance mechanisms, treatment outcomes, systematic review, meta-analysis

## Abstract

**Background**: Bedaquiline (BDQ) resistance poses a serious threat to its long-term efficacy, particularly in high-burden settings like South Africa, where data remain scattered and largely non-synthesized. **Objective**: This study aimed to estimate the trends of BDQ resistance in drug resistant tuberculosis (DR-TB), characterize associated resistance mechanisms, and evaluate implications for treatment outcomes in South Africa. **Eligibility criteria**: We included primary studies reporting BDQ resistance, resistance mechanisms, minimum inhibitory concentrations (MICs), or treatment outcomes among patients with MDR- or XDR-TB treated with BDQ-containing regimens in South Africa. **Information sources**: PubMed, Web of Science, and Embase were searched for studies published between January 2016 and July 2024. **Risk of bias**: Study quality was assessed using the Joanna Briggs Institute (JBI) Critical Appraisal Checklist. **Synthesis of results:** Random-effects meta-analysis with Freeman–Tukey double-arcsine transformation was used to estimate pooled BDQ resistance prevalence. Heterogeneity, sensitivity analyses, and publication bias were assessed. **Results:** Twenty-eight studies were included. The pooled prevalence of BDQ resistance was 6.0% (95% CI: 4.1–7.9%; I^2^ = 62%). Treatment success averaged 63.5%, and culture conversion reached 84.1%. Resistance-associated mutations were most frequently reported in *Rv0678*, followed by *atpE* and *pepQ*, often associated with elevated MICs (≥2–4 μg/mL). Evidence of small-study effects was observed (Egger’s test, *p* = 0.0012). A pooled prevalence estimate was calculated; however, evidence of small-study effects suggests that estimates should be interpreted cautiously. **Limitations:** Heterogeneity in study design, outcome definitions, and resistance testing methods limited comparability across studies. **Conclusions:** Bedaquiline remains effective for DR-TB treatment in South Africa; however, emerging resistance and its molecular drivers pose a growing threat to regimen sustainability, including BPaL. Strengthened surveillance and standardized resistance testing are urgently needed.

## 1. Introduction

Bedaquiline (BDQ), a novel diarylquinoline, represents a landmark advancement in tuberculosis (TB) therapeutics as the first new anti-tuberculosis drug to be introduced in more than four decades. Bedaquiline has rapidly become a cornerstone in the management of multidrug-resistant tuberculosis (MDR-TB), offering renewed hope for improved outcomes. However, the recent emergence of BDQ resistance threatens to undermine its clinical effectiveness and global TB control efforts [1,2]. In South Africa, BDQ was first introduced in 2012 under a compassionate use program, reflecting the urgent need for new treatment options for MDR-TB. Its demonstrated efficacy led to its subsequent addition to the WHO-recommended all-oral standard regimens worldwide for patients with MDR-TB. Today, BDQ stands serves as a cornerstone of global DR-TB treatment strategies [3].

South Africa was among the first countries to adopt the use of BDQ, extending its use beyond its initial role as a therapeutic option to managing toxicity, most notably the hearing loss that occurs with TB injectable drugs [4]. When BDQ was approved for inclusion in the national TB program in 2013, it signaled a transformative moment in TB care, offering a ground-breaking mechanism of action that redefined treatment possibilities [4,5]. BDQ acts by providing a novel mechanism of action which inhibits mycobacterial ATP synthase, blocking ATP production, which is vital for the survival of mycobacteria [6]. Bedaquiline has presented a substantial advancement in increasing positive treatment outcomes and decreasing mortality among MDR and extensively drug-resistant TB (XDR-TB) patients [7,8,9,10].

However, this therapeutic advantage is increasingly being considerably undermined by the alarming rates at which BDQ resistance is being reported in South Africa and beyond [11,12]. Resistance to the drug will not only reduce its effectiveness but also threatens to make current TB treatment programs non-viable. Factors contributing to BDQ resistance include poor drug stewardship, late detection of resistance, and the inherent adaptive biological ability of *Mycobacterium tuberculosis* (*M. tuberculosis*). Bedaquiline resistance in *M. tuberculosis* is predominantly mediated through genetic mutations that affect either drug target binding or intracellular drug concentration. The most frequently reported mechanism involves mutations in *Rv0678*, a transcriptional regulator of the MmpL5–MmpS5 efflux pump, leading to increased efflux of bedaquiline and reduced drug susceptibility. Less commonly, resistance arises from mutations in *atpE*, which encodes the ATP synthase subunit targeted by bedaquiline, as well as mutations in *pepQ* which have been associated with low-level resistance. These mechanisms underpin both acquired resistance during treatment and the transmission of resistant strains [6,13]. The escalating prevalence of BDQ resistance in South Africa presents a critical breeding ground for further resistance evolution, thereby threatening the success of current TB control efforts.

Understanding emerging resistance is particularly important because BDQ may likely continue playing a critical role in the global response to TB through the BPaL regimen [14]. Resistance to BDQ threatens to undermine public health efforts aimed at containing DR-TB. The South African experience highlighted in this review offers valuable lessons to the global community, emphasizing the need of vigilance in resistance monitoring, investing in a strong diagnostic system, and evidence-based interventions to secure a future for BDQ. Although BDQ has improved DR-TB outcomes, data on emerging resistance in South Africa remain fragmented due to heterogeneity in study designs, provincial coverage, outcome definitions, and inconsistent reporting of resistance mechanisms and MIC thresholds.

Despite the widespread programmatic use of bedaquiline in South Africa and increasing reports of resistance, evidence remains fragmented, heterogeneous, and unevenly reported across provinces and study designs. Most available studies are observational, vary in resistance definitions, and often lack standardized reporting of molecular mechanisms or minimum inhibitory concentrations, limiting comparability and interpretation. Furthermore, no prior systematic review or meta-analysis has comprehensively synthesized bedaquiline resistance prevalence, the associated genetic mechanisms, and clinical outcomes within the South African context. Given South Africa’s early adoption of bedaquiline and its central role in emerging regimens such as bedaquiline–pretomanid–linezolid (BPaL), a rigorous synthesis of resistance trends and mechanisms is urgently needed to inform treatment policy, surveillance strategies, and stewardship efforts. Thus, the objective of this study is to systematically review and meta-analyze available evidence on bedaquiline resistance in South Africa, characterize underlying resistance mechanisms, and assess their clinical and public health implications.

## 2. Results

### 2.1. Identification of Relevant Studies

**Figure 1:**
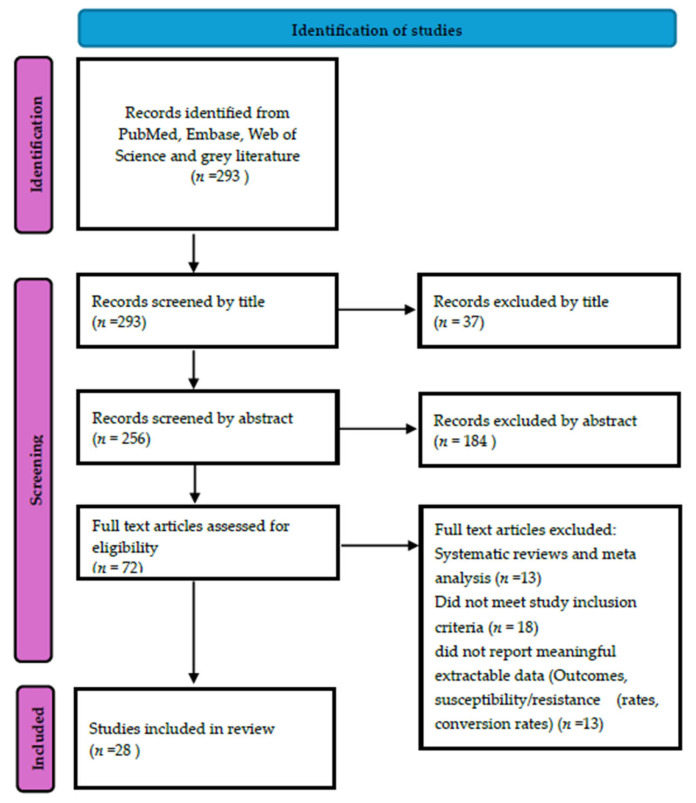
PRISMA flow diagram of study selection for systematic review of bedaquiline resistance in South Africa.

After removing duplicates (*n* = 51), 293 unique records were screened. The systematic search yielded 293 non-duplicated records from PubMed and Web of Science. At the title screening stage, 37 records were excluded, and a further 184 were excluded after abstract review. Seventy-two full-text articles were further assessed for eligibility. Some articles were excluded due to being systematic reviews or meta-analyses (*n* = 13), lacking extractable outcome data (*n* = 13), or not meeting the inclusion criteria (*n* = 18). Ultimately, 28 studies were deemed eligible and included in the final systematic review. The detailed study selection process is illustrated in the PRISMA flow diagram in Figure 1.

### 2.2. Summary of Characteristics of Included Studies

Treatment outcomes, mortality rates, BDQ resistance, and culture conversion varied notably across provinces and scopes. Gauteng reported the highest average treatment success at 90% (95% CI: 85–94), with low mortality (6–12%) and minimal BDQ resistance (~1%, 95% CI: 0–3), alongside excellent culture conversion (~90%, 95% CI: 87–93). In contrast, KwaZulu-Natal showed the lowest treatment success at 57% (95% CI: 52–62), with elevated mortality (15–20%) and the highest average BDQ resistance (~10.3%, 95% CI: 7–14). The Western Cape demonstrated moderate treatment success (70%, 95% CI: 65–75) and resistance (~7.6%, 95% CI: 5–10), with strong culture conversion (~85–90%, 95% CI: 82–92). Multi-province and nationally scoped studies revealed intermediate outcomes, with treatment success ranging from 59% to 72%, mortality between 14% and 24%, and BDQ resistance averaging 8.5–11.2% (95% CI: 7–13). The Eastern Cape, represented by a single study, reported 62% treatment success and 21% mortality, with no resistance or culture conversion data available. Overall, across all 28 studies, the pooled treatment success was 63.5% (95% CI: 59–68), mortality averaged 14.7% (95% CI: 12–17), BDQ resistance was 6.0% (95% CI: 4.1–7.9), and culture conversion reached 84.1% (95% CI: 80–88). These findings underscore regional disparities in BDQ performance and highlight the need for targeted interventions in provinces with lower success and higher resistance rates. (Table 1).

Mutations associated with BDQ resistance were described in 43% of studies, most frequently involving *Rv0678*, *atpE*, and *pepQ*; these were reported in numerous studies, often in conjunction with MICs exceeding 1.0–4.0 μg/mL. (Table 2).

### 2.3. Reporting Quality and Completeness of Included Studies

The methodological quality of the included studies was systematically assessed using the Joanna Briggs Institute (JBI) Critical Appraisal Checklist for Cohort Studies. A total of 28 included studies were evaluated across eight quality domains. The majority of the studies (*n* = 24; 85.7%) achieved a score of 8/8, indicating high methodological quality, achieving full scores across all criteria. Three studies (10.7%) scored between 6 and 7 across domains with “Yes” responses, reflecting moderate quality, while one study (3.6%) scored ≤5, indicating low quality. Common limitations among moderate- and low-quality studies included inadequate identification and management of confounding variables, unclear strategies for managing confounding, and incomplete or unclear reporting of statistical procedures (Table 3). Overall, the evidence base was dominated by high-quality studies, supporting the robustness of the review’s findings. Despite these isolated weaknesses, the overall evidence base was characterized by strong internal validity and consistent adherence to appraisal standards, reinforcing the reliability of the pooled findings and subgroup analyses presented in this review.

### 2.4. Pooled Estimates of Treatment Outcomes and Resistance Rates

A total of 28 studies were included in this review, reporting on clinical and microbiological outcomes associated with BDQ treatment for DR-TB. Among the 20 studies that reported treatment success rates, the unweighted mean was 63.5%, while the weighted mean, accounting for sample size, was slightly higher at 65.5%, reflecting generally favorable outcomes across diverse programmatic and clinical settings. Twelve studies provided data on BDQ resistance rates with sample sizes; the unweighted mean was 7.6%, while the weighted pooled prevalence was 6.0% (95% CI: 4.0–8.1%), indicating that larger studies generally reported lower resistance levels.

### 2.5. Subgroup Analyses by Geographic Region and Year of Publication

Subgroup analyses were conducted to explore variation in treatment success and BDQ resistance rates by geographic region and year of publication. Treatment success rates varied across provinces. The highest average was reported in Gauteng (90.0%), followed by studies conducted across multiple provinces (approximately 72.3%), the Western Cape (70.2%), and the Eastern Cape (62.0%). Lower success rates were observed in KwaZulu-Natal (55.8%) and nationally aggregated studies (59.5%). BDQ resistance rates also varied by location. The highest average resistance rate was observed in multi-province studies (11.2%), followed by KwaZulu-Natal (10.3%), national-level studies (8.5%), the Western Cape (7.6%), and Gauteng (1.0%).

When analyzed by year of publication, treatment success rates peaked in 2020 (74.1%) and 2021 (73.0%), while slightly lower averages were recorded in 2018 (65.6%) and 2022 (64.2%). A notable decline was observed in 2023 (27.3%), primarily due to a small-sample study reporting 0% success. BDQ resistance rates remained relatively low between 2018 and 2020 (5.0–5.2%), followed by an increase in 2022 (10.3%) and 2023 (20.0%). The 2023 value was notably influenced by a small cohort study [35] reporting 20% resistance. Heterogeneity was moderate among studies published between 2018 and 2020 (I^2^ = 53.1%, 95% CI: 0.0–90.3%), while heterogeneity was negligible in studies published between 2021 and 2024 (I^2^ = 0.0%, 95% CI: 0.0–87.9%). The wide confidence intervals reflect the relatively small number of studies in each subgroup.

### 2.6. Trends in Treatment Success Rates and BDQ Resistance

**Figure 2:**
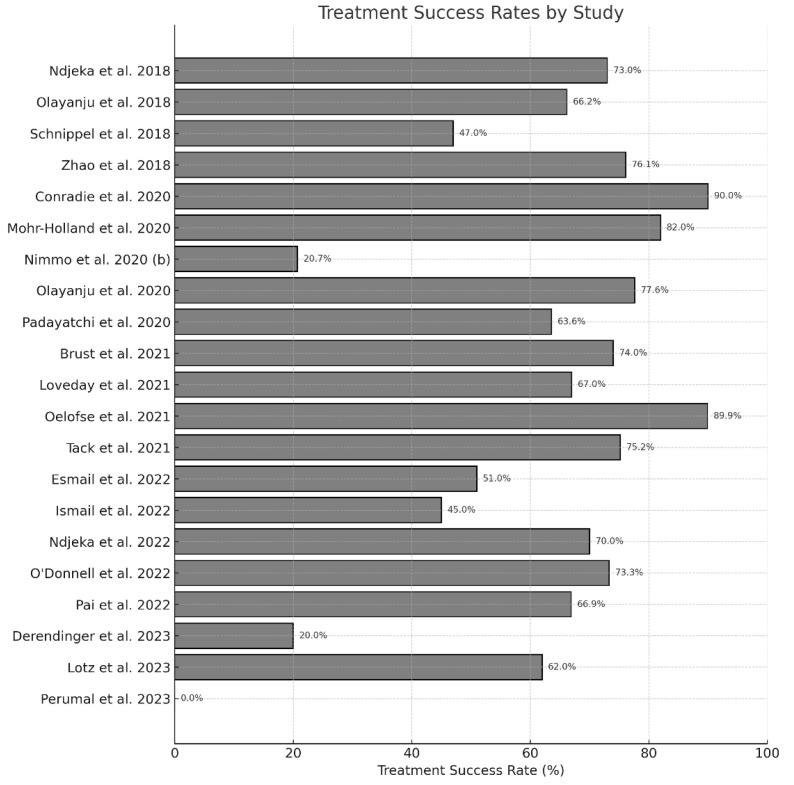
BDQ treatment success rates by study [7,9,11,13,16,17,19,24,25,26,27,28,29,30,31,32,33,34,35].

**Figure 3:**
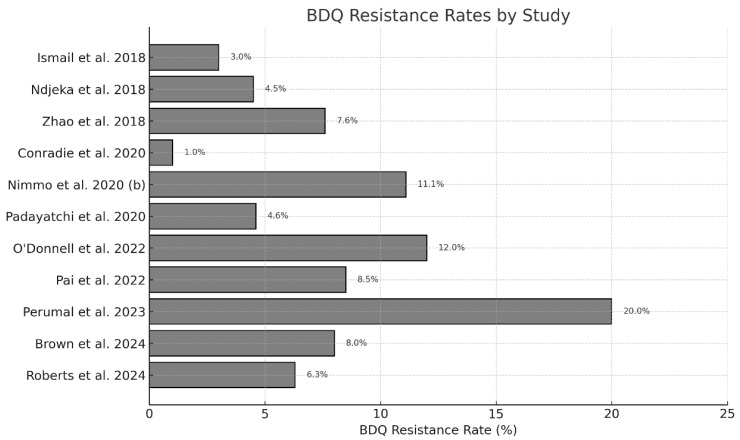
BDQ resistance rates by study [9,15,16,19,20,21,22,29,30,35,36,37].

Treatment success rates peaked in 2020 and 2021 (averaging around 74% and 73%, respectively), reflecting improved clinical management during this period. When stratified by year of publication, unweighted treatment success rates varied across years and appeared lower in 2023. However, this pattern was driven by a single small-sample study (*n* = 5) reporting 0% treatment success. When weighted by sample size, treatment success estimates remained stable over time, with no evidence of a population-level decline. Specifically, the unweighted mean treatment success in 2023 was 27.3%, whereas the corresponding weighted mean was 63.1%, consistent with pooled estimates from larger cohorts (Figure 2). Weighted analysis showed BDQ resistance remained low from 2018 to 2020 (around 5.11%) but increased to approximately 10% in 2022 and 20% in 2023 (Figure 3). These trends suggest sustained treatment effectiveness overall, but highlight emerging concerns around rising resistance that warrant continued monitoring. The 2023 increase was influenced by smaller studies reporting higher resistance proportions, underscoring potential emerging challenges with BDQ resistance in more recent cohorts. Overall, while treatment success remains generally favorable, the upward trajectory in BDQ resistance rates emphasizes the need for ongoing surveillance and targeted interventions to prevent resistance development.

### 2.7. Trends in BDQ Resistance Mechanisms and Highest Reported MICs

Trends in BDQ resistance mechanisms revealed that mutations in *Rv0678* were the most commonly reported, identified in approximately 75% of the studies that analyzed genotypic resistance. Mutations in *atpE* were less frequent, reported in around 25% of those studies, while *pepQ* mutations were noted in about 15%. These mutations were often linked to elevated BDQ MICs, typically ranging from >0.25 to 4.0 μg/mL. In accordance with WHO recommendations, an MIC ≥1.0 μg/mL was used as the critical concentration for defining phenotypic resistance to bedaquiline, while higher MIC values reflect increasing or high-level resistance as reported in the primary studies. MIC thresholds varied, with some studies reporting intermediate resistance (e.g., MICs of 0.5–1.0 μg/mL), and others documenting high-level resistance (≥2.0–4.0 μg/mL). *Rv0678* mutations alone were associated with a wide MIC range, indicating variability in phenotypic resistance. In contrast, higher MICs (≥4.0 μg/mL) were more often linked to mutations in *atpE* or concurrent mutations in multiple resistance-associated genes. These findings underscore the genetic diversity underlying BDQ resistance and highlight the importance of combining molecular and phenotypic methods for accurate detection and interpretation.

### 2.8. Meta-Analysis of BDQ Resistance Rates

**Figure 4:**
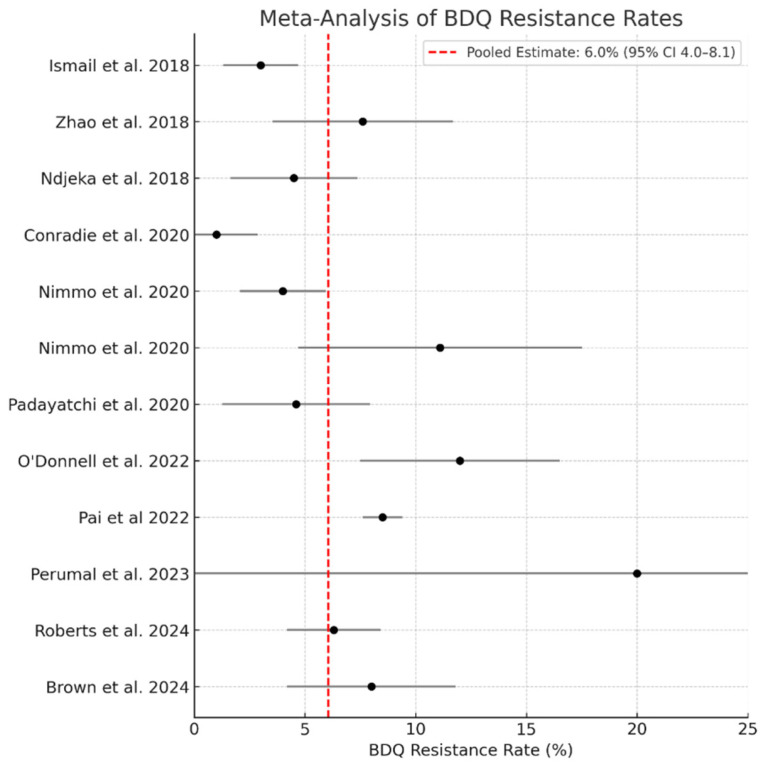
Forest Plot of BDQ Resistance Rates and Pooled Estimate Across Studies [9,15,16,19,20,21,22,29,30,35,36,37].

Meta-analysis was conducted using a random-effects model with Freeman–Tukey double-arcsine transformation to estimate the pooled prevalence of BDQ resistance across 11 studies reporting both resistance rates and sample sizes. Exploratory temporal analyses suggested a higher frequency of reported bedaquiline resistance in more recent publication periods. The pooled BDQ resistance rate was 6.0% (95% CI: 4.1–7.9%), with substantial heterogeneity (I^2^ = 62.3%, 95% CI: 22.8–87.8%; τ^2^ = 0.00939). The 95% prediction interval ranged from approximately 1.2% to 17.8%, indicating considerable variability in resistance prevalence that may be expected across future settings. A forest plot of individual study estimates and confidence intervals is presented in Figure 4. These analyses are intended to be hypothesis-generating and should be interpreted cautiously given the limited number of contributing studies and absence of longitudinal denominators.

### 2.9. Publication Bias Analysis

**Figure 5:**
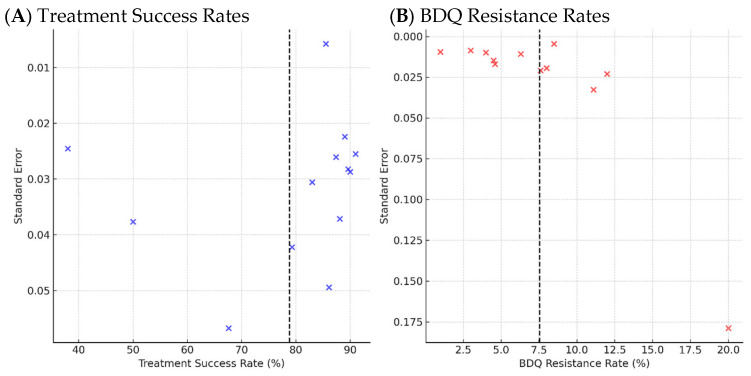
Funnel Plots Assessing Publication Bias for Treatment Success [9,15,16,19,20,21,22,29,30,35,36,37] and BDQ Resistance Rates [9,15,16,19,20,21,22,29,30,35,36,37]. (**A**) Treatment success rate: a symmetrical distribution suggests minimal publication bias. (**B**) BDQ resistance rate: visible asymmetry driven by an outlier study suggests possible reporting bias or small-study effects.

The funnel plot for treatment success demonstrated a relatively symmetrical distribution of effect sizes around the mean, indicating minimal evidence of publication bias across studies. In contrast, the funnel plot for BDQ resistance rates showed marked asymmetry, largely driven by a single small-sample study reporting an unusually high resistance rate of 20%. This pattern suggests potential small-study effects or selective reporting influencing BDQ resistance estimates. These visual findings highlight the need for cautious interpretation of pooled BDQ resistance estimates (Figure 5).

### 2.10. Sensitivity Analysis

**Figure 6:**
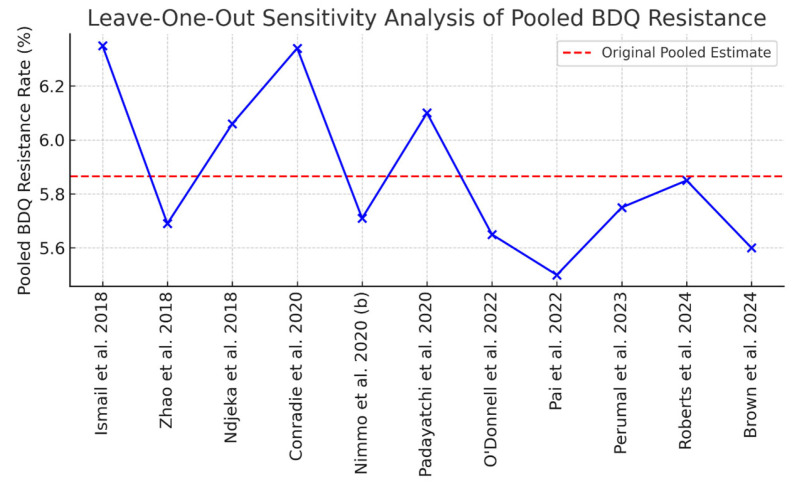
Influence plot for leave-one-out sensitivity analysis [9,15,16,19,20,21,22,29,30,35,36,37].

A leave-one-out sensitivity analysis demonstrated that the pooled bedaquiline resistance estimate was stable, varying modestly between 5.46% and 6.35% upon exclusion of individual studies (overall estimate: 6.0%). Although the pooled prevalence estimate remained numerically stable across sensitivity analyses, the presence of statistically significant small-study effects and funnel plot asymmetry indicates that the pooled estimate should be interpreted cautiously. Larger studies, such as [30]; *n* = 3747), exerted greater influence on the pooled estimate, while the exclusion of small-sample studies (e.g., [35]) resulted in minimal changes. The influence plot (Figure 6) indicates that no single study substantially altered the overall estimate.

Sensitivity analyses excluding case reports and studies with very small cohorts yielded pooled estimates comparable to the primary analysis.

**Figure 7:**
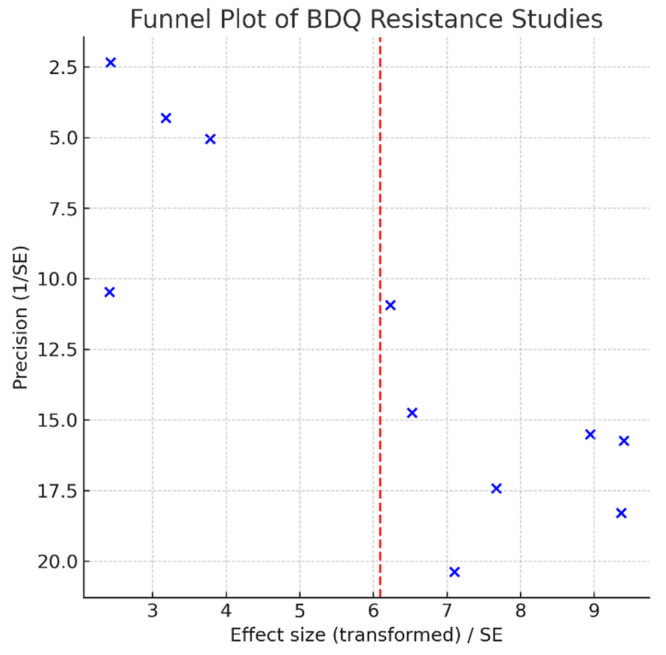
Funnel plot assessing publication bias in BDQ resistance studies using transformed effect size (Freeman–Tukey double-arcsine transform).

Assessment of small-study effects using a funnel plot of transformed effect sizes revealed asymmetry (Figure 7). Egger’s test indicated significant asymmetry (*p* = 0.0012).

Stratified meta-analysis by publication year revealed a statistically significant increase in pooled BDQ resistance rates between studies published in 2018–2020 and those published in 2021–2024 (4.3% [95% CI 2.7–6.4%] vs. 7.8% [95% CI 6.1–9.7%]; *p* = 0.0004). Heterogeneity was moderate in the earlier period (I^2^ = 53.1%), whereas statistical heterogeneity was low in the later period (I^2^ = 0%). However, the latter finding should be interpreted cautiously, as it may reflect the small number of included studies rather than true clinical homogeneity. Leave-one-out sensitivity analyses within each stratum produced narrow pooled ranges (2018–2020: 3.77–4.82%; 2021–2024: 7.44–8.74%) (Table 4). These findings are consistent with an upward temporal trend in reported BDQ resistance and support cautious interpretation and strengthened surveillance going forward.

### 2.11. Implications of Results for Future BDQ Use in South Africa

These findings have important implications for the continued use of BDQ in South Africa. While treatment success rates are moderate overall (typically ~67–70%) and vary widely across cohorts (45–90%), the gradual increase in BDQ resistance, particularly in recent years, warrants prospective monitoring and careful interpretation of health system outcomes. The pooled resistance rate of 6.0% suggests emerging resistance mutations and regional variability, highlighting the need for ongoing surveillance, appropriate regimen design, and expanded access to resistance testing to preserve its utility in DR-TB treatment. Strengthening diagnostic capacity and standardizing reporting will be critical to preserve BDQ’s utility in DR-TB treatment and to safeguard the effectiveness of newer regimens such as BPaL.

## 3. Discussion

This systematic review provides the most comprehensive synthesis to date of BDQ treatment outcomes and resistance patterns in South Africa. Bedaquiline, the backbone of all-oral DR-TB regimens, has improved outcomes while reducing toxicities linked to injectable agents. Our pooled analysis confirms that BDQ has transformed DR-TB therapy, with culture conversion rates exceeding 80% and treatment success averaging 63.5%. Yet resistance estimated at 6% overall and exceeding 10% in certain provinces raises urgent concerns about the sustainability of these gains. To date, systematic reviews of BDQ treatment outcomes and resistance in South Africa remain limited, despite reports of emerging resistance [11,18] and BDQ’s widespread implementation [23,32]. This study addresses that gap by consolidating evidence on culture conversion, treatment success, resistance rates, and underlying resistance mechanisms.

This study reported consistency in weighted vs. unweighted means in treatment success and resistance rates in South Africa. These findings demonstrate overall promising treatment outcomes but also underscore the need for continued resistance monitoring. The consistency between unweighted and weighted means suggests less influence from small-study effects on pooled estimates. However, between-study variability, particularly in reporting formats and population characteristics, reinforces the importance of subgroup analyses and cautious interpretation when comparing results across settings and time periods. While South Africa was among the earliest adopters of BDQ under programmatic conditions, this study demonstrates the urgent need to preserve BDQ’s effectiveness.

Culture conversion rates provide an important early indicator of regimen effectiveness and mirror many of the provincial patterns observed in treatment outcomes. Across the 28 included studies, overall culture conversion was high at 84.1% (95% CI: 80–88%), consistent with the expected benefits of BDQ-containing regimens. Yet the aggregated figures mask notable provincial disparities. Gauteng achieved the highest conversion (~90%), reflecting strong diagnostic capacity and timely regimen management. Western Cape followed closely (~85–90%), aligning with its moderate resistance levels and relatively stable program performance. KwaZulu-Natal showed lower conversion (~79–83%), consistent with its higher HIV/TB co-infection burden, elevated BDQ resistance, and greater vulnerability to delays in diagnosis and treatment optimization. Data from the Eastern Cape were too limited for reliable estimation, underscoring persistent provincial information gaps. Consistent with other reports [38,39,40] these patterns suggest that while BDQ has improved bacteriological clearance nationally, sustained gains remain closely tied to provincial health system capacity, HIV epidemiology, and access to rapid diagnostics. Interpretation of provincial-level differences is limited by substantial heterogeneity across provinces, including differences in HIV prevalence, referral and case-mix patterns, diagnostic and sequencing capacity, study designs, and study periods. These factors introduce confounding and selection bias, precluding direct epidemiological comparisons between provinces. Accordingly, provincial findings should be interpreted as descriptive summaries of reported evidence rather than comparative estimates of resistance burden.

The emergence of BDQ resistance represents a critical threat to regimen sustainability. Molecular studies have identified mutations in *Rv0678*, *atpE*, and *pepQ* as key drivers of resistance, often associated with elevated minimum inhibitory concentrations (MICs ≥ 2–4 µg/mL). These mutations alter efflux pump regulation and ATP synthase binding, reducing BDQ’s bactericidal activity [41]. Importantly, resistance has been detected not only in patients with prior BDQ exposure but also in those receiving BDQ for the first time, suggesting transmission of resistant strains [11]. Provincial disparities mirror these biological findings: Gauteng’s minimal resistance (~1%) reflects strong stewardship and routine molecular surveillance, while KwaZulu-Natal’s higher rates (~10%) highlight the vulnerability of high-burden settings with limited diagnostic capacity. The Western Cape’s intermediate resistance (~7–8%) underscores the need for sustained monitoring even in provinces with relatively strong infrastructure. These patterns echo international reports, where BDQ resistance has been documented in 4–10% of patients, reinforcing that this is not a localized phenomenon but a global challenge [42,43] The observation of MICs ≥4.0 µg/mL in a subset of isolates is consistent with high-level bedaquiline resistance and may reflect cumulative resistance-conferring mutations reported in prior studies. Although exploratory temporal analyses suggested higher reported bedaquiline resistance in more recent periods, these findings should not be interpreted as confirmatory evidence of a national increase in resistance. The resistance meta-analysis was based on a limited number of studies, predominantly small cohorts and programmatic datasets from selected provinces. Apparent temporal increases may reflect intensified surveillance, expanded access to drug susceptibility testing, or increased reporting rather than true population-level trends. Taken together, these findings emphasize that BDQ resistance is shaped by both microbiological adaptation and programmatic weaknesses, underscoring the urgent need for genomic surveillance, rapid diagnostics, and stewardship practices to preserve BDQ’s long-term efficacy.

Our pooled analysis highlights both treatment success and mortality patterns, underscoring the uneven impact of BDQ across South African provinces. Gauteng reported the strongest outcomes, with ~90% treatment success, low mortality (6–12%), and minimal BDQ resistance (~1%), consistent with Ndjeka et al. (2022), who documented improved survival following the rollout of all-oral regimens in urban centers with robust laboratory infrastructure and patient monitoring [32]. In contrast, KwaZulu-Natal showed markedly poorer success (~57%), higher mortality (15–20%), and elevated resistance (~10%), echoing findings from Ismail et al. (2018) and Omar et al. (2022), where BDQ resistance and high TB/HIV co-infection rates were linked to adverse outcomes [15,44] The Western Cape demonstrated intermediate success (~70%), resistance (~7–8%), and mortality (14–18%), while nationally scoped studies reported averages of 59–72% success and 14–24% mortality, reinforcing that BDQ’s survival benefit is not evenly distributed [16,23,24]. The Eastern Cape, with success at 62% and mortality exceeding 20% in the single study available, highlights the vulnerability of provinces with limited surveillance data [34]. Our subgroup analysis by publication period demonstrated moderate heterogeneity in earlier studies and lower statistical heterogeneity in more recent reports. Although statistical heterogeneity was negligible in the 2021–2024 subgroup, this should be interpreted cautiously. The I^2^ statistic has limited power when few studies are included and may underestimate true underlying clinical and programmatic heterogeneity, particularly in complex TB settings spanning multiple provinces.

Several factors likely explain the provincial disparities observed in our pooled analysis. Provinces with stronger laboratory infrastructure and routine drug susceptibility testing detect resistance earlier and adjust regimens more effectively, while regions with higher HIV prevalence, such as KwaZulu-Natal, face compounded risks of poor immune recovery, drug–drug interactions, and immune reconstitution inflammatory syndrome [45,46] Delays in treatment initiation, interruptions in drug supply, and inconsistent access to companion drugs undermine regimen durability, and socioeconomic barriers such as poverty, malnutrition, and limited transport reduce adherence and follow-up [47]. Differences in patient support systems, including adherence counseling, psychosocial support, and community health worker coverage, further influence survival outcomes [48]. Taken together, these figures suggest that while BDQ has reduced mortality compared to older regimens, its impact is strongly mediated not only by pharmacological efficacy but also by provincial health system capacity, HIV/TB co-epidemics, diagnostic access, and broader programmatic and structural determinants [49]. Overall, while South Africa’s pooled outcomes with BDQ fall within global ranges, the sharp provincial contrasts highlight the importance of context in sustaining treatment success. Gauteng’s high success and minimal resistance demonstrate what is achievable with strong infrastructure, while KwaZulu-Natal’s poorer outcomes and elevated resistance underscore the vulnerability of high-burden, resource-constrained settings. The Western Cape’s intermediate results and the Eastern Cape’s limited data further illustrate heterogeneity across the country. By situating South Africa as both a sentinel and a cautionary case, these findings reinforce the need for region-specific interventions and provide lessons for the continent and global TB community as they strive to meet WHO End TB Strategy targets.

These findings have direct implications for the implementation and stewardship of newer regimens, including bedaquiline, pretomanid, and linezolid (BPaL). As BDQ is the cornerstone of this all-oral, shortened regimen, emerging resistance threatens not only current DR-TB treatment models but also the future scalability of BPaL. Routine resistance testing prior to initiating BDQ-based regimens, though not yet widely available, should be prioritized in high-burden settings where BDQ exposure is common. In the absence of baseline susceptibility data, the use of BPaL could inadvertently amplify resistance and compromise treatment success. South Africa’s experience provides an early warning for the global TB community. As one of the first high-burden countries to scale up BDQ nationally, South Africa offers a unique window into both the benefits and risks of widespread use. The sharp provincial disparities demonstrate how BDQ’s effectiveness is highly dependent on health system capacity, HIV/TB co-epidemics, and programmatic stewardship. The emergence of resistance in KwaZulu-Natal and the Western Cape, despite BDQ being relatively new, signals that resistant strains can arise rapidly and may spread before routine diagnostics are in place. This trajectory mirrors earlier experiences with fluoroquinolones and injectables, where early gains were eroded by resistance due to gaps in stewardship. Globally, many countries are now adopting BDQ-based regimens, including BPaL, without the same level of surveillance infrastructure that South Africa has begun to implement. If resistance is already detectable in South Africa, a country with comparatively strong laboratory networks and early adoption, the risk in settings with weaker systems is even greater. Recognizing South Africa as a sentinel case highlights the urgency of embedding pharmacovigilance, standardized MIC testing, molecular resistance markers, and equitable health system strengthening into global TB strategies before resistance undermines the promise of BDQ and novel regimens. At the national level, strengthening laboratory infrastructure, expanding rapid molecular diagnostics, integrating HIV and TB services, ensuring uninterrupted drug supply chains, and investing in patient support systems are critical to sustain BDQ’s gains.

Region-specific stewardship strategies are required to address provincial disparities, with Gauteng serving as a model of best practice and KwaZulu-Natal and the Eastern Cape highlighting areas of vulnerability. Internationally, BDQ’s promise can only be safeguarded through coordinated surveillance, stewardship, and integration of social determinants into TB care. By aligning these interventions with WHO End TB Strategy targets of ≥90% treatment success and reduced mortality, South Africa can consolidate BDQ’s progress and provide lessons for the global fight against drug-resistant TB.

While BDQ remains an indispensable component of DR-TB treatment in South Africa and beyond, its long-term effectiveness hinges on a coordinated response that balances access with resistance mitigation. Policymakers and TB control programs must act decisively to integrate resistance monitoring into clinical practice, adapt treatment algorithms based on emerging evidence, and safeguard the future of novel regimens like BPaL in the global fight against DR-TB.

## 4. Materials and Methods

### 4.1. Study Design

The review was conducted and reported in accordance with the PRISMA 2020 guidelines, and the completed PRISMA checklist is provided in Supplementary File S2. It aims to assess the prevalence, mechanisms, and clinical implications of BDQ resistance among individuals treated for MDR or XDR-TB in South Africa from January 2016 to July 2024. The protocol for this systematic review was submitted to PROSPERO prior to completion of data extraction and analysis and was assigned the registration number CRD420251124914. No amendments were made to the information provided in the registration. The timeframe (January 2016 to July 2024) was selected to coincide with the introduction and programmatic scale-up of BDQ-containing regimens for drug-resistant tuberculosis in South Africa. Bedaquiline was recommended by the World Health Organization in 2016 and subsequently adopted into national DR-TB treatment guidelines, making studies published before this period less relevant for assessing resistance patterns, molecular mechanisms, and treatment outcomes associated with routine clinical use.

### 4.2. Eligibility Criteria

We included studies of patients with MDR/XDR-TB treated with a BDQ-containing regimen in South Africa. Outcomes reporting BDQ resistance rates, resistance mechanisms, minimum inhibitory concentration (MIC), and treatment outcomes such as cure or mortality were included. Eligible study designs included observational studies (cohort, case–control, cross-sectional), clinical trials, case series, and case reports. We restricted inclusion to English-language publications between January 2016 and July 2024. Although all included studies met the eligibility criteria for inclusion in this review, not all reported complete data for every outcome of interest. In particular, only a subset of studies provided detailed information on bedaquiline resistance mechanisms, minimum inhibitory concentrations, or culture conversion, which limited the scope of certain subgroup and comparative analyses. Case reports and small cohort studies were included to capture early and emerging evidence of bedaquiline resistance, which remains relatively uncommon and is often first detected in individual cases or small programmatic cohorts. Excluding these studies would risk underestimating resistance emergence and omitting clinically relevant resistance mechanisms, particularly in high-burden settings such as South Africa.

### 4.3. Information Sources and Search Strategy

In accordance with Cochrane recommendations, a comprehensive literature search was conducted across multiple databases, including PubMed, Web of Science, and Embase. To minimize publication bias, gray literature was also searched using Google Scholar, conference proceedings, and reference lists of included studies. We developed a comprehensive search strategy using a combination of Medical Subject Headings (MeSH), Embase subject headings (Emtree), and free-text keywords. Key terms included “Bedaquiline”, “Tuberculosis”, “drug-resistant tuberculosis”, “Mycobacterium tuberculosis”, “South Africa”, “resistance mechanisms”, “treatment outcomes”, “systematic review”, and “meta-analysis”. Boolean operators and database-specific controlled vocabulary were adapted for each database.

### 4.4. Study Selection

The search results were imported into Rayyan literature review software (Version 1.4.3) screening (https://www.rayyan.ai/ (accessed on 7 February 2025). Title and abstract screening was performed independently by two reviewers with expertise in TB. Disagreements were resolved through discussion between the two reviewers, and where necessary adjudicated by a third reviewer. Full texts of potentially eligible articles were retrieved and assessed using a standardized eligibility form. In total, 28 studies were included. The study selection process is presented in a PRISMA flow diagram.

### 4.5. Data Extraction

A pre-piloted data extraction form was used by two independent reviewers. Extracted information included study characteristics (authors, publication year, province, study design, and sample size), BDQ resistance metrics (prevalence, mutation types), MIC values, and treatment outcomes (e.g., cure rate, culture conversion, mortality). Discrepancies or unclear entries were resolved by contacting the corresponding authors or excluding the study if unresolved.

### 4.6. Quality Assessment and Risk of Bias

The methodological quality and risk of bias of included studies were assessed using standardized criteria appropriate for their study designs. Study quality and risk of bias were assessed using the Joanna Briggs Institute (JBI) Critical Appraisal Checklists appropriate to each study design. JBI appraisal results were used to inform qualitative interpretation of the evidence and to contextualize findings, rather than as a basis for statistical weighting or study exclusion. All studies meeting eligibility criteria were retained in the analyses regardless of JBI score. Two reviewers independently assessed each study, and discrepancies were resolved through discussion or consultation with a third reviewer. Key domains evaluated included participant selection, measurement of exposures and outcomes, confounding, and statistical analysis. Studies were categorized as having low, moderate, or high risk of bias based on overall scores. The results of the quality assessments were used to inform the interpretation of the findings and the strength of the evidence base.

### 4.7. Statistical Analysis and Data Synthesis

Statistical analyses were conducted using IBM SPSS Statistics (version 30.0; IBM Corp., Armonk, NY, USA). Pooled estimates of BDQ resistance, culture conversion rates, and treatment success rates with corresponding 95% confidence intervals (CIs) were estimated using either a fixed-effect or random-effects model, with the random-effects model (restricted maximum likelihood) applied in most cases due to anticipated variability in true effect sizes across studies. For proportions, the Freeman–Tukey double-arcsine transformation was used to stabilize variances before pooling.

Between-study heterogeneity was assessed using Cochran’s Q statistic, the I^2^ statistic, and τ^2^. The I^2^ statistic was interpreted according to established thresholds, with values of approximately 25%, 50%, and 75% representing low, moderate, and high heterogeneity, respectively. To improve interpretability and reproducibility, 95% confidence intervals (CIs) for I^2^ were calculated based on the non-central chi-square distribution of the Q statistic. Where substantial heterogeneity was detected (I^2^ > 50%), subgroup and sensitivity analyses were undertaken to explore potential sources of variability. Confidence intervals for I^2^ were calculated using the chi-square-based method described by Higgins and Thompson, with Cochran’s Q reconstructed where necessary. Sensitivity analyses were performed using leave-one-out (study influence) diagnostics to evaluate the robustness of pooled estimates. Meta-analyses were not weighted by JBI scores; inverse-variance weighting was applied based on study precision.

Publication bias was assessed using Egger’s regression test to detect small-study effects (*p* < 0.05 indicating statistical significance) and visual inspection of funnel plots for asymmetry. Where indicated, the potential impact of small-study effects on pooled estimates was considered.

A narrative synthesis was undertaken to summarize observed trends in BDQ resistance, resistance mechanisms, regional differences, and associated clinical outcomes. Provincial-level analyses were conducted descriptively to summarize the geographic distribution of reported resistance and were not intended for formal inter-provincial comparison. For outcomes with at least 10 studies, a random-effects meta-analysis was performed to estimate pooled prevalence, with heterogeneity and publication bias reassessed in the final model.

The certainty of evidence for the primary outcome (pooled BDQ resistance prevalence) was assessed using the GRADE approach. As the included studies were predominantly observational, the certainty of evidence was initially rated as low and further evaluated based on risk of bias, inconsistency, indirectness, imprecision, and publication bias. Overall certainty of evidence was judged to be low, primarily due to between-study heterogeneity and evidence of small-study effects.

## 5. Conclusions

This systematic review and meta-analysis addressed the research question by quantifying the prevalence and temporal trends of bedaquiline resistance in South Africa, identifying key genetic mechanisms associated with resistance, and contextualizing these findings in relation to treatment outcomes. The pooled analysis suggests that while bedaquiline-containing regimens remain effective for DR-TB, bedaquiline resistance is present at a measurable level and exhibits regional and temporal variability; however, this estimate should be interpreted cautiously given between-study heterogeneity and variability in resistance definitions. Mutations in *Rv0678*, *atpE*, and *pepQ* were the most frequently reported resistance-associated genetic determinants. Collectively, these findings inform the sustainability of bedaquiline-based and BPaL regimens and underscore the need for strengthened resistance surveillance, standardized reporting, and routine susceptibility testing to preserve treatment efficacy.

## 6. Strengths and Limitations

This study’s strengths include its comprehensive scope across provinces, integration of clinical and molecular data, and consistency between weighted and unweighted pooled estimates, which reduces concerns about small study effects. By disaggregating outcomes provincially, it highlights heterogeneity often masked in national averages and provides policy-relevant insights for stewardship.

The limitations of this study lie in the heterogeneity in study designs, populations, and outcome definitions, reducing the comparability of findings and precluding formal meta-analysis for all outcomes. Many studies lacked standardized reporting on BDQ resistance mechanisms, MIC thresholds, or follow-up duration, which may have led to underestimation or misclassification of resistance. First, only a subset of studies provided detailed data on culture conversion, resistance mutations, or treatment outcomes, reducing the robustness of subgroup analyses. Second, some resistance estimates were derived from small sample sizes, increasing the risk of sampling bias and limiting generalizability. Third, the absence of consistent genotypic or phenotypic testing across studies may have led to variability in resistance detection and reporting. As the review was restricted to studies conducted in South Africa, the findings may not be directly generalizable to other settings with different epidemiological or programmatic contexts. Resistance definitions varied across studies, including phenotypic susceptibility testing, genotypic mutation-based definitions, or a combination of both. Pooling these heterogeneous definitions into a single resistance outcome may have introduced misclassification and should be interpreted as an overall programmatic estimate rather than a strictly standardized resistance prevalence.

Most included studies were conducted in only three provinces, resulting in limited representation from other regions of South Africa. This geographic imbalance restricts the generalizability of the findings at a national level. This review included only English-language publications, which may have resulted in the exclusion of relevant studies published in other languages. To mitigate this, we conducted comprehensive searches across multiple international databases and screened reference lists of included studies; however, some non-English studies may still have been missed.

Inclusion of case reports and small cohorts may increase variance and introduce small-study effects; however, this approach was necessary to comprehensively capture emerging bedaquiline resistance and was mitigated through random-effects modeling and sensitivity analyses.

Publication bias is a potential limitation of this review. The funnel plot for BDQ resistance showed asymmetry, supported by a significant Egger’s test, suggesting small-study effects or selective reporting, with smaller studies tending to report higher resistance rates; therefore, the pooled resistance estimate should be interpreted with caution.

Although the protocol was registered with PROSPERO, formal confirmation occurred after initiation of data extraction. No deviations from the registered protocol were made.

## Figures and Tables

**Table 1:** Summary of Characteristics of Included Studies (2018–2024).

Province/Scope	No. of Studies	Avg. Treatment Success (95% CI)	Avg. Mortality (95% CI)	Avg. BDQ Resistance (95% CI)	Avg. Culture Conversion (95% CI)
Western Cape	8	70% (65–75%)	8–13% (6–15%)	~7.6% (5–10%)	~85–90% (82–92%)
KwaZulu-Natal	7	57% (52–62%)	15–20% (12–23%)	~10.3% (7–14%)	~79–83% (75–86%)
Gauteng	3	90% (85–94%)	6–12% (5–14%)	~1% (0–3%)	~90% (87–93%)
Eastern Cape	1	62% (55–68%)	21% (17–25%)	Not reported	Not reported
Multi-province/National	9	59–72% (55–75%)	14–24% (12–27%)	~8.5–11.2% (7–13%)	~84% (80–88%)
**Overall (28 studies)**	**28**	**63.5% (59–68%)**	**14.7% (12–17%)**	**6.0% (4.1–7.9%)**	**84.1% (95% CI: 80–88%)**

**Table 2:** Characteristics of studies included in systematic review.

Study	Year	Location	Sample Size	BDQ Culture Conversion Rate	BDQ Resistance Rate	Highest BDQ MIC Observed	Resistance Mechanisms Identified (Mutations)	Treatment Success Rate	Mortality Rate
Ismail et al. [15]	2018	South Africa	391	38%	3%	4 µg/mL	*Rv0678, pepQ, Rv1979c*	-	-
Zhao et al. [16]	2018	Western Cape	162	87.4%	7.6%	-	-	76.1%	6.7%
Olayanju et al. [7]	2018	Western Cape	68	67.6%	-	-	-	66.2%	14.7%
Schnippel et al. [17]	2018	South Africa	1016	-	-	-	-	47%	12.6%
Ndjeka et al. [9]	2018	Gauteng, KwaZulu-Natal, Northern Cape, North West and Western Cape	200	-	4.5%	-	-	73%	12.5%
Ismail et al. [18]	2019	Gauteng	-	-	-	>2 μg/mL	*Rv0678, atpE*	-	-
Conradie et al. [19]	2020	Gauteng	109	90%	1%	>2 μg/mL	*Rv0678*	90%	6%
Nimmo et al. [20]	2020	KwaZulu-Natal	391	-	4%	1.0 µg/mL	*Rv0678*	-	-
Nimmo et al. [21]	2020	KwaZulu-Natal	92	79.3%	11.1%	1.0 µg/mL	*Rv0678*	-	20.7%
Padayatchi et al. [22]	2020	KwaZulu-Natal	151	83%	4.6%	-	-	63.6%	17.2%
Olayanju et al. [23]	2020	Western Cape	76	88.1%	-	-	-	77.6%	2.7%
Mohr-Holland et al. [24]	2020	Western Cape	126	91%	-	-	-	82%	8%
Loveday et al. [25]	2021	KwaZulu-Natal	108	-	-	-	-	67%	7%
Tack et al. [26]	2021	KwaZulu-Natal	117	89.6%	-	-	-	75.2%	12.8%
Brust et al. [27]	2021	KwaZulu-Natal & Western Cape	195	89%	-	-	*Rv0678*	74%	13%
Oelofse et al. [28]	2021	Western Cape, KwaZulu-Natal, Gauteng	211	-	-	-	-	89.9%	6.4%
O’Donnell et al. [29]	2022	KwaZulu-Natal	199	-	12%	1.0 µg/mL	-	73.3%	16.1%
Pai et al. [30]	2022	South Africa	3747	85.5%	8.5%	-	-	66.9%	15.4%
Esmail et al. [31]	2022	South Africa	49	86.1%	-	-	-	51%	8.2%
Ndjeka et al. [32]	2022	South Africa	688	-	-	-	-	70%	24%
Ismail et al. [13]	2022	South Africa	176	50%	-	-	*Rv0678, atpE, mmpL5*	45%	38%
Rivière et al. [33]	2022	Western Cape	509	-	-	0.5 µg/mL	*atpE, Rv0678, Rv0676c, Rv0677c, pepQ and Rv1979c*	-	-
Mohr-Holland et al. [24]	2022	Western Cape	2008	-	-	-	-	-	8%
Lotz et al. [34]	2023	Eastern Cape	282	-	-	-	-	62%	21.3%
Perumal et al. [35]	2023	KwaZulu-Natal	5	-	20%	4 µg/mL	*Rv0678, Rv1979c*	0%	-
Derendinger et al. [11]	2023	Western Cape	40	-	-	-	*Rv0678, atpE, pepQ, Rv0676c, Rv0677c, and Rv1979c*	20%	50%
Roberts et al. [36]	2024	Gauteng and Western Cape	505	-	6.3%	-	*mmpR5, mmpL5, mmpL3, mmpS5, atpE, amiA2, pepQ, era, rv1816, and rv3249c.*	-	-
Brown et al. [37]	2024	South Africa	195	-	8%	>4 µg/mL	*mmpR5*	-	-

**Table 3:** Quality assessment of included studies using the JBI Critical Appraisal Checklist.

Study	Year	Q1	Q2	Q3	Q4	Q5	Q6	Q7	Q8	Total “Yes”
Ismail et al. [15]	2018	Y	Y	Y	Y	Y	Y	Y	Y	**8**
Zhao et al. [16]	2018	Y	Y	Y	Y	Y	Y	Y	Y	**8**
Olayanju et al. [7]	2018	Y	Y	Y	Y	Y	Y	Y	Y	**8**
Schnippel et al. [17]	2018	Y	Y	Y	Y	Y	Y	Y	Y	**8**
Ndjeka et al. [9]	2018	Y	Y	Y	Y	Y	N	Y	Y	**7**
Ismail et al. [18]	2019	Y	Y	Y	Y	U	U	Y	N/A	**5**
Conradie et al. [19]	2020	Y	Y	Y	Y	Y	Y	Y	Y	**8**
Nimmo et al. [20]	2020	Y	Y	Y	Y	Y	Y	Y	Y	**8**
Nimmo et al. [21]	2020	Y	Y	Y	Y	Y	N	N	Y	**6**
Padayatchi et al. [22]	2020	Y	Y	Y	Y	Y	Y	Y	Y	**8**
Olayanju et al. [23]	2020	Y	Y	Y	Y	Y	Y	Y	Y	**8**
Mohr-Holland et al. [24]	2020	Y	Y	Y	Y	Y	Y	Y	Y	**8**
Loveday et al. [25]	2021	Y	Y	Y	Y	Y	Y	Y	Y	**8**
Tack et al. [26]	2021	Y	Y	Y	Y	Y	Y	Y	Y	**8**
Brust et al. [27]	2021	Y	Y	Y	Y	Y	Y	Y	Y	**8**
Oelofse et al. [28]	2021	Y	Y	Y	Y	Y	Y	Y	Y	**8**
O’Donnell et al. [29]	2022	Y	Y	Y	Y	Y	Y	Y	Y	**8**
Pai et al. [30]	2022	Y	Y	Y	Y	Y	Y	Y	Y	**8**
Esmail et al. [31]	2022	Y	Y	Y	Y	Y	Y	Y	Y	**8**
Ndjeka et al. [32]	2022	Y	Y	Y	Y	Y	Y	Y	Y	**8**
Ismail et al. [13]	2022	Y	Y	Y	Y	Y	Y	Y	Y	**8**
Rivière et al. [33]	2022	Y	Y	Y	Y	Y	Y	Y	Y	**8**
Mohr-Holland et al. [24]	2022	Y	Y	Y	Y	Y	Y	Y	Y	**8**
Lotz et al. [34]	2023	Y	Y	Y	Y	Y	Y	Y	Y	**8**
Perumal et al. [35]	2023	Y	Y	Y	Y	U	U	Y	Y	**6**
Derendinger et al. [11]	2023	Y	Y	Y	Y	Y	Y	Y	Y	**8**
Roberts et al. [36]	2024	Y	Y	Y	Y	Y	Y	Y	Y	**8**
Brown et al. [37]	2024	Y	Y	Y	Y	N	N	Y	N	**5**

Y = Yes (criterion met), N = No (criterion not met), and U = Unclear (insufficient information). Keys: Q1 Were the criteria for inclusion in the sample clearly defined? Q2 Were the study subjects and the setting described in detail? Q3 Was the exposure measured in a valid and reliable way? Q4 Were objective, standard criteria used for measurement of the condition? Q5 Were confounding factors identified? Q6 Were strategies to deal with confounding factors stated? Q7 Were the outcomes measured in a valid and reliable way? Q8 Was appropriate statistical analysis used?

**Table 4:** BDQ resistance: pooled rates, heterogeneity and leave-one-out ranges by year group (Freeman–Tukey transform; random effects).

Year Group	Studies (n)	Pooled Resistance	95% CI (%)	I^2^ (%)	τ^2^	Total Events
2018–2020	7	4.31	2.65–6.35	53.1	0.00631	48
2021–2024	5	7.79	6.09–9.68	0.00	0.00000	65

Overall pooled estimate (all studies combined): 6.0% (95% CI 4.15–7.86%), I^2^ = 62.3%.

## Data Availability

All data are available within the manuscript text.

## Supplementary Materials

The following supporting information can be downloaded at: https://www.mdpi.com/article/10.3390/antibiotics15040385/s1, File S1: Search strategy; File S2: PRISMA_2020_checklist [50]; Table S1: GRADE Summary of Findings.
