# Supplementary material for: Bedaquiline Resistance in Drug-Resistant Tuberculosis in South Africa: A Systematic Review and Meta-Analysis of Emerging Trends
Source: Antibiotics (Basel). 2026 Apr 10;15(4):385. doi: 10.3390/antibiotics15040385 (PMC13114208; doi:10.3390/antibiotics15040385)
Supplement: Supplementary file 1 [file antibiotics-15-00385-s001.zip › Supplementary file S1 Search strategy.pdf]

PubMed Seach strategy

("Bedaquiline"[MeSH] OR bedaquiline)

AND

("Tuberculosis, Multidrug-Resistant"[MeSH] OR "drug-resistant tuberculosis" OR MDR-TB OR XDR-TB)

AND

("South Africa"[MeSH] OR "South Africa")

Filters: Publication date from 2016/01/01 to 2024/07/31; English

Embase Search strategy

('bedaquiline' OR bedaquiline)

AND

('drug resistant tuberculosis' OR 'multidrug resistant tuberculosis')

AND

('south africa' OR 'south africa’)

Limits: 2016–2024; English

Web of Science Search Strategy

TS=(bedaquiline AND ("drug-resistant tuberculosis" OR MDR-TB OR XDR-TB) AND "South Africa")

Timespan: 2016–2024
