# Supplementary material for: Bedaquiline Resistance in Drug-Resistant Tuberculosis in South Africa: A Systematic Review and Meta-Analysis of Emerging Trends
Source: Antibiotics (Basel). 2026 Apr 10;15(4):385. doi: 10.3390/antibiotics15040385 (PMC13114208; doi:10.3390/antibiotics15040385)
Supplement: Supplementary file 1 [file antibiotics-15-00385-s001.zip › Supplementary table S1 GRADE.pdf]

Supplementary Table S1: GRADE Summary of Findings

Outcome	No. of studies	Study design	Risk of bias	Inconsistency	Indirectness	Imprecision	Publication bias	Certainty
BDQ resistance prevalence	11	Observational	Not serious	Serious	Not serious	Not serious	Likely	**Low**
